# Artificial Intelligence Technologies in Cardiology

**DOI:** 10.3390/jcdd10050202

**Published:** 2023-05-06

**Authors:** Łukasz Ledziński, Grzegorz Grześk

**Affiliations:** Department of Cardiology and Clinical Pharmacology, Faculty of Health Sciences, Collegium Medicum in Bydgoszcz, Nicolaus Copernicus University in Toruń, Ujejskiego 75, 85-168 Bydgoszcz, Poland; 503345@doktorant.umk.pl

**Keywords:** artificial intelligence, cardiology, machine learning

## Abstract

As the world produces exabytes of data, there is a growing need to find new methods that are more suitable for dealing with complex datasets. Artificial intelligence (AI) has significant potential to impact the healthcare industry, which is already on the road to change with the digital transformation of vast quantities of information. The implementation of AI has already achieved success in the domains of molecular chemistry and drug discoveries. The reduction in costs and in the time needed for experiments to predict the pharmacological activities of new molecules is a milestone in science. These successful applications of AI algorithms provide hope for a revolution in healthcare systems. A significant part of artificial intelligence is machine learning (ML), of which there are three main types—supervised learning, unsupervised learning, and reinforcement learning. In this review, the full scope of the AI workflow is presented, with explanations of the most-often-used ML algorithms and descriptions of performance metrics for both regression and classification. A brief introduction to explainable artificial intelligence (XAI) is provided, with examples of technologies that have developed for XAI. We review important AI implementations in cardiology for supervised, unsupervised, and reinforcement learning and natural language processing, emphasizing the used algorithm. Finally, we discuss the need to establish legal, ethical, and methodical requirements for the deployment of AI models in medicine.

## 1. Introduction

There is a growing need to find a novel approach for using the enormous amounts of data that are collected every day for all aspects of life. According to the International Data Corporation (IDC), 153 exabytes of healthcare data were produced in 2013; it has been estimated that 2314 exabytes of healthcare data will have been produced in 2020 and that with this astronomical rate of growth, healthcare data will soon reach the zettabyte scale [1]. The healthcare industry has always generated huge quantities of information, and digital transformation has already begun. Information gathered from patients is often stored in electronic format—electronic health records (EHR)—and medical test results, imaging results, clinical notes, and in-patient interviews are being digitalized [2]. The volume of healthcare data is growing exponentially—not only data acquired by healthcare professionals (HCPs), but also data obtained by wearable sensors and via applications created to improve therapy adherence. This growing volume of data could be characterized as "4Vs”—high volume, high velocity, high veracity, and high variety—which makes data very difficult to manage using simple methods. Artificial intelligence (AI)—the computer of computers to mimic human intelligence—has significant potential to impact a complex domain such as medicine. AI is likely to be the basis for a significant revolution in healthcare systems. It has already had an immense impact on molecular chemistry and drug discovery. The techniques used in the prediction of molecules’ pharmacological activities are often based on artificial neural networks [3,4,5]. The possibility of using in silico experiments is crucial for achieving reductions in costs and time. Currently, there is considerable scientific pressure to develop new drugs that will be more effective, applicable to new pathogens, and easier to administer. Furthermore, in silico experiments are much more in line with “green chemistry” than traditional experiments. There is even an established consortium, the Machine Learning for Pharmaceutical Discovery and Synthesis Consortium (MLPDSC), which includes the Massachusetts Institute of Technology (MIT) and representatives of 13 pharmaceutical and chemical companies [6,7,8].

As a discipline, AI includes systems that act like human intelligence—AI can learn and make decisions. Growing curiosity about artificial intelligence started in the 1950s when Alan Turing asked if machines could think and John McCarthy used the phrase “artificial intelligence” for the first time [9,10]. Now, thanks to improvements in hardware and affordability, interest in AI is increasing and it is being developed. In 2015, AlphaGo—a program based on a deep learning algorithm—defeated a human professional Go player for the first time in history [11]. 

The first big subpart of AI is machine learning (ML), which has the capability to developing systems that learn from retrospective data and achieve, as a result, classifications, clustering, or regression models. Algorithms of ML can detect patterns in complex data. Such a feature can be helpful in interpreting results and making personalized clinical decisions. Unlike traditional statistics, which are focused on creating scoring systems, machine learning develops automated clinical decision systems [12,13,14,15,16,17,18]. ML is already being used to create screening and diagnostic models [19,20,21,22,23].

Cardiology is a complex field of medicine that focuses on the diagnosis, treatment, and management of cardiovascular diseases. Due to its complexity, the diagnosis and treatment of cardiovascular diseases can be challenging. Cardiovascular diseases include a wide range of conditions that affect the heart and blood vessels, such as coronary artery disease, heart failure, arrhythmia, and valvular heart disease. These diseases can cause a range of symptoms, including chest pain, shortness of breath, fatigue, dizziness, and many deviations in laboratory parameters.

Medical imaging techniques, such as echocardiography, cardiac MRI, and CT scans, are among the primary diagnostic tools used in cardiology. These imaging methods provide detailed information about the heart’s structure and function, enabling clinicians to detect abnormalities and diagnose cardiovascular diseases. Cardiac catheterization is another diagnostic procedure that is used to assess the heart’s blood vessels and the blood flow to and from the heart.

The treatment of cardiovascular diseases varies, depending on the type and severity of the condition. Treatment options range from lifestyle modifications, such as diet and exercise, to medications, surgery, and medical devices such as pacemakers and implantable cardioverter-defibrillators (ICDs). Cardiac rehabilitation programs may also be recommended to help patients recover after a cardiac event and to improve their overall cardiovascular health.

Cardiology analysis differs from the analysis of other areas of the body, due to the heart’s unique anatomy and physiology. The heart is a dynamic organ that constantly adapts to changes in the body’s demand for oxygen and nutrients. The heart’s intricate system of valves, chambers, and blood vessels requires specialized expertise to interpret and diagnose cardiovascular diseases accurately.

In recent years, AI technologies have been increasingly used in cardiology to improve the accuracy and efficiency of diagnosis, treatment, and management of cardiovascular diseases. AI-based image analysis algorithms can quickly and accurately detect abnormalities in medical images, and predictive models can analyze vast amounts of patient data to identify patterns and predict the likelihood of developing certain cardiovascular diseases. AI-based decision support systems can also assist clinicians in making treatment decisions, and wearable devices can monitor heart health and detect early signs of cardiovascular disease. With ongoing developments in AI technologies, the field of cardiology is poised to continue making significant advances in the diagnosis and management of cardiovascular diseases, ultimately leading to better patient outcomes.

The focus of this review is on the application of artificial intelligence technologies in the field of cardiology, in contrast to most of the similar reviews that concentrate on the use of AI in cardiology fields. The potential benefits of AI in cardiology are vast, including improved diagnostic accuracy, risk prediction, and treatment outcomes. Machine learning algorithms, neural networks, and natural language processing techniques are among the AI technologies that have been applied in cardiology. However, there are also challenges to overcome, including data privacy and security concerns, regulatory and legal issues, and the need for effective communication between AI systems and healthcare providers. The successful implementation and integration of AI into clinical practice require extensive testing and validation to ensure safety and effectiveness.

In the first part of the paper, general assumptions of AI are presented. Then, focus is placed on individual technologies and their potential applications in cardiology are described. Examples of works that use some of the aforementioned techniques are presented, followed by a discussion highlighting the challenges for the further implementation of artificial intelligence.

## 2. Introduction to AI in Healthcare

Obtaining a model by machine learning algorithms is preceded by the defining of the objective, data gathering, data preparation, data exploration. splitting data, and training using big data sets derived from many sources. The next step after training is tuning the hyperparameters, proceeding with testing, and validating. 

The first step in the ML process is describing the problem to be faced. It is necessary to know what type of input data are available, which features are targeted, and of the nature of the problem. This step is followed by the most time-consuming and difficult element—gathering data from electronic health records and data curation by considering missing values, unnecessary information, etc. Exploratory data analysis is crucial in understanding the correlations, associations, patterns, and trends in the data.

**Figure 1:**
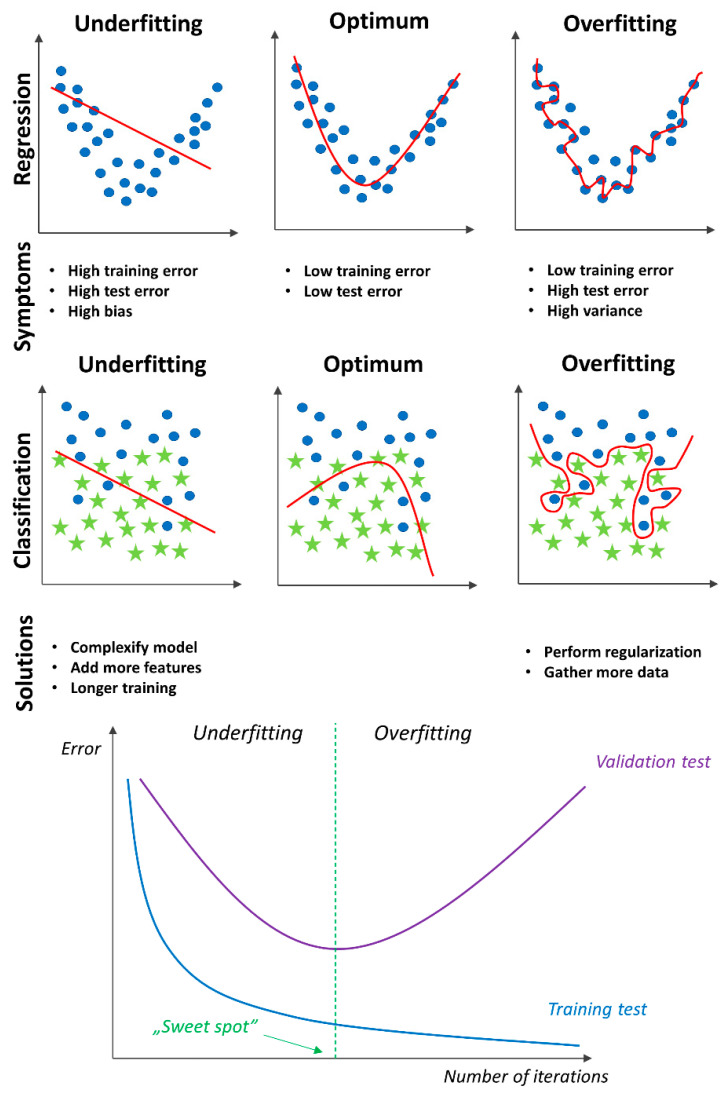
Underfitting and overfitting examples.

**Figure 2:**
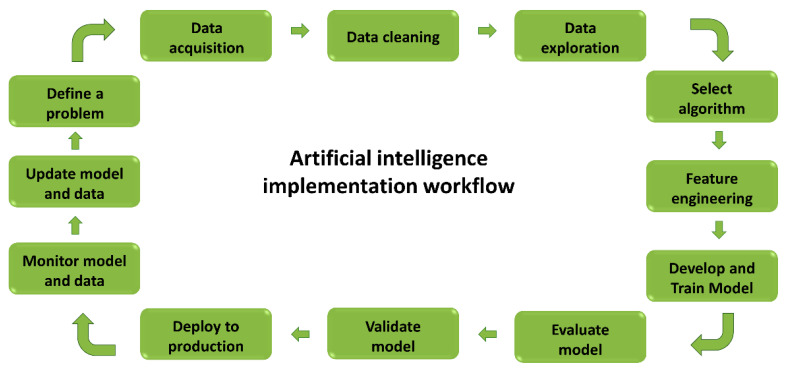
Artificial intelligence workflow.

Before beginning to build a model, it is essential to understand that there are three main types of ML: supervised learning, unsupervised learning, and reinforcement learning. Supervised learning uses labeled data with a known outcome that is of interest for training set and for generating a model that adjusts weights until the model is fit for classification or regression. Classification analysis focuses on a matching class for each object; for example, in medicine, it can be used to check whether or not a patient has a disease. In a short term, the output of a classification model is usually nominal. On the other hand, regression models usually have continuous output that, for example, could be used in medicine to estimate the likelihood of disease. The learning process must be followed by cross-validation to ensure that the model is not underfitting or overfitting. An underfitting model will not be able to accurately predict or classify unseen data and training data; overfitting occurs when a model is ideal for training data but cannot perform accurately against unknown data [24]. See Figure 1. An artificial intelligence implementation workflow is presented on Figure 2.

Unsupervised learning uses unlabeled data as the input for analyzing and clustering by taking advantage of patterns that are present in the shared commonality of the data—a method that is widely used in medicine. Reinforcement learning (RL) might be described as the machine learning method for processing dynamic data, adapting an algorithm to change and optimize to achieve the best possible result. The learning is based on trial and error to achieve the maximum reward.

## 3. The Most-Often-Used ML Algorithms

### 3.1. Supervised Learning

1. K-nearest neighbors (KNN)

KNN is a classification algorithm that is focused on finding similarity. The basic idea is to find a group of similar objects in a dataset and make predictions based on majority voting. KNN has two prominent hyperparameters to tune—distance function and K value. Determining the value of K is done by trial. The main issue is a scaling problem, which is usually solved by feature engineering.

2. Decision trees (DTs)

DTs are among the simplest models that are related to classification and regression. A decision tree is an algorithm that is presented with the tree structure, which splits data multiple times according to a specified threshold in the variables on the feature which, when split, provides the highest information gain. By division, different subsets of the dataset, called internal nodes, are created. Final subsets are called terminal leaves. An ensembled learning algorithm based on a decision tree is random forest (RF). In RF, many trees are created using random subsets of features and bootstrapped data; then, each tree votes by predicting a target class and all votes are tallied to reach the final prediction. The main advantage of RF is better accuracy without a higher computational workload. Another example of an ensemble decision-tree model is the gradient-boosted tree (GBT). Like RF, it can be used for solving a classification and regression problem by combining the outputs from individual trees. The main difference is in the way the individual trees are built and how the results are calculated. For RF purposes, bagging is implemented; on the other hand, GBT uses boosting, a technique that fits the sequence of models by adding higher weights to records, with higher residuals in each correct match. In other words, each new tree corrects the errors of the previous one, improving efficiency, accuracy, and interpretability. GBT has even higher accuracy than RF, but comes with a higher computational cost.

3. Support Vector Machines (SVMs)

The goal of SVMs is to delineate the hyperplanes that separate observations into two separate classes, with a maximum margin. SVMs were developed for linear classification, thanks to the development of kernel functions for nonlinear spaces. The choice of kernel functions must be based on existing data.

4. Naïve Bayes (NB)

Bayes’ rule provides a formula for determining the probability of Y by a given X. When functions are independent, the Bayes rule can be extended to the so-called naïve Bayesian rule. Naïve Bayes is a classification technique that uses the most basic knowledge of probability and makes the naïve assumption that all characteristics are independent. An algorithm tries to find probabilities using known preliminary probabilities that are learned from training data. NB models are fast in computing and have worked quite well in solving problems such as spam filtering.

5. Artificial Neural Networks (ANNs)

Artificial neural networks simulate the behavior of the human brain and humanlike abilities to learn. The architecture of a neural network refers to the units, their activation functions, and the number of layers that may be found through experimentation. A single unit of an ANN is the neuron, which has an input value, weights and bias, a net sum, and an activation function. Inputs are multiplied by weights, summed along with a bias, then transformed by an activation function. Neural nets are trained by iterations with an optimalization function. After each training cycle, called an epoch, an error metric is calculated based on the difference between the prediction and the goal. The derivatives of this error metric are computed and propagated back through the network using a technique called back-propagation. The factors (weights) of each neuron are then adjusted, depending on how much they contributed to the total error. This process is repeated iteratively until the network error falls below an acceptable threshold. Neurons are aggregates of layers—input layers—that collect initial data and send them further (each neuron from the input layer sends data to a neuron in the hidden layer), and hidden layers, which are intermediate states. Initially, the process of learning and searching for linear and nonlinear relationships is carried out. There may be multiple hidden layers (deep learning). The more hidden layers a network has, the more deeply it can find dependencies. Finally, the output layer (the result) is returned.

### 3.2. Unsupervised Learning

1. K-means

K-means algorithms are a prominent example of grouping algorithms. The five steps in a K-means algorithm can be distinguished. The first step is the selection of the number of clusters. Then, K starting points are drawn into the space (K first centroids). The third step is the mapping of all observations to the nearest centroid, using a given distance measure (the Euclidean distance), followed by finding a new centroid for each of the K-groups. The drawing and mapping steps are repeated until the stop condition is reached—for example, reaching the point where the assignment of observations to groups does not change, or reaching the assumed number of iterations. The main advantages of a K-means algorithm are simplicity and speed of operation. However, the disadvantages are the need to determine the number of groups, the sensitivity to the selection of the starting points, and the sensitivity to the impact of outliers.

2. Principal Component Analysis (PCA)

Principal component analysis is perhaps the most popular dimension-reduction algorithm. In a nutshell, it consists of projecting data to a space with a smaller number of dimensions in order to best preserve the data structure. It is primarily used to reduce the variables that describe a given phenomenon and to discover possible regularities among the features. A thorough analysis of principal components enables the identification of those initial variables that have a large impact on the appearance of individual principal components, i.e., those variables that make up a homogeneous group. It is worth remembering that, with PCA, we lose some information and we also lose interpretability.

3. Hierarchical clustering

Hierarchical clustering, as the name suggests, is an algorithm that builds a hierarchy of clusters. Hierarchical agglomeration clustering starts with all data points that are assigned to one’s own cluster. Then, the two closest clusters are combined into the same cluster. The algorithm ends when there is only one remaining cluster. The algorithm merges groups in such a way that the variance inside them is as low as possible and the variance among the groups is as high as possible. These algorithms represent a detail-to-general approach. The approach implemented in the agglomeration algorithms works well when we decide to search for small groups (after several iterations, the process may be interrupted). The result of hierarchical clustering can be presented as a dendrogram. Hierarchical deglomeration clustering works the other way around, i.e., we start with one group and divide it into smaller groups in subsequent iterations.

4. A priori algorithms

A priori algorithms are well-known for mining association rules. They use frequent item sets for generating those rules and finding common patterns in a database. A priori algorithms are based on a “bottom-up” approach—i.e., frequent subsets are extended one at the time and then tested. The termination of the algorithm occurs when no further successful extension can be made. The main advantage of this algorithm is its ability to establish a relationship among events.

Healthcare AI applications are more likely to be based on supervised learning, while unsupervised learning is used for pre-processing dimension reductions, clustering, or finding relationships among observations [25,26]. Perhaps this usage of supervised learning in healthcare results from the enhanced explainability of supervised models [27]. There are high concerns about the safety, responsibility, and reliability of AI-based systems; a great deal of emphasis is placed on explainable artificial intelligence (XAI). A big warning that applies to a blind trust in AI was evident in the errors made by IBM Watson. a well-known algorithm used by many hospitals around the world, which recommended treatment for patients with cancer. As was pointed out by oncologists, the training dataset was unrealistic and too biased, and was also labeled by only few specialists instead of by guidelines or evidence-based medicine (EBM), which yielded many erroneous recommendations made by the algorithm [28,29]. It should be highlighted that medical malpractice is usually harmful for only a few patients, but a flawed AI model is a vast risk for a multitude of patients. XAI provides general information on how AI makes its decisions by disclosing the strengths and weaknesses of a program, why the model makes a specific decision, and specific criteria on the basis of which the program undertakes decisions. To overcome this explainability challenge, many novel technologies are being developed [30,31,32].

XAI employs a wide range of techniques to enhance the transparency and interpretability of machine learning algorithms. These techniques include:

1. Rule-based systems: Rule-based systems are among the earliest techniques used in XAI and they are still widely used today. These systems rely on predefined rules and decision trees that explicitly state how an algorithm makes decisions. This approach allows humans to trace the decision-making process, making it more transparent and understandable.
2. Model interpretation techniques: Model interpretation techniques are used to provide insight into how the machine learning model makes predictions. These techniques include visualization tools that help users understand the model’s internal structure and feature importance analysis that shows which features are most important for the model’s decisions.
3. Local explanations: Local explanations aim to provide an explanation for a single prediction. These techniques are used to explain why the machine learning algorithm made a specific decision for a given input. Examples of local explanation methods include LIME (local interpretable model–agnostic explanations) and SHapley additive exPlanations (SHAP).
4. Global explanations: Global explanations aim to provide explanations for the overall behavior of the machine learning model. These techniques include sensitivity analysis, which shows how changes in input features affect a model’s output, and feature importance analysis, which shows which features are most important for a model’s overall performance.
5. Human-in-the-loop: Human-in-the-loop (HITL) techniques involve incorporating human feedback into the machine learning process. These techniques allow humans to provide input and feedback at various stages of the model development process, increasing transparency and improving the model’s performance.
6. GradCAM: gradient-weighted class activation mapping (GradCAM) is a visualization technique that highlights the regions of an image that are most important for the machine learning algorithm’s decision-making process. GradCAM works by generating a heatmap that shows the areas of an image that had the highest activation in the convolutional layers of the deep neural network used by the machine learning model.

To compare a few models, it is necessary that some metrics show how good a model is in solving a problem. The most popular performance metrics are summarized in Table 1 and Table 2.

## 4. Natural Language Processing

An important feature of artificial intelligence, which is widely applied in medicine, is natural language processing (NLP). NLP is the capability of automatically analyzing and representing human language using computational methods. The variety of possibilities gained by applying NLP to medicine includes text classification, information extraction, and search engines for the effective use of clinical notes. Another aspect of natural language processing is the use of speech recognition and question-answering to develop chatbots for patients [33]. Combining NLP and ML methods provides an opportunity to extract valuable resources from a large amount of unstructured EHR data that may be used in preparing clinical decision-making support systems, diagnostic tools, or recommendation systems based on search engines applied to scientific papers.

There are six main fazes of NLP:

1. Morphological analysis

Morphological analysis deals with understanding separate words according to their morphemes. We refer to the morpheme as the smallest unit of meaning.

2. Lexical analysis

Lexical analysis involves identifying and analyzing the structure of words and dividing an entire text into paragraphs, sentences, and words. To deal with lexical analysis, we often need to normalize lexicons.

3. Syntactic analysis

Syntax analysis is used to evaluate the compliance of a language with grammatical rules. Computer algorithms are used to apply grammatical rules to a group of words and to derive meanings from those words.

4. Semantic analysis

Semantic analysis is the use of computer algorithms to understand the meaning and interpretation of words and the structures of sentences. This is done by mapping syntactic structures and objects in the sentence domain. In other words, semantic analysis focuses on the interactions among meanings at the word level in a sentence. The best-known technique for semantic analysis is unit name recognition (NER).

5. Discourse integration

Discourse integration is concerned with how an immediately preceding sentence can influence the interpretation of the following sentence. Discourse integration focuses on the properties of the text, as a whole, that convey meaning by creating connections among sentences. In other words, it is focused on acquiring context from other sentences.

6. Pragmatic analysis

Pragmatic analysis explains how additional meaning is loaded into texts without actually being encoded into them. This requires a great deal of knowledge about the world, including an understanding of intentions, plans, and goals.

## 5. The implementation of Artificial Intelligence in Cardiology

### 5.1. Implementation of AI Technologies in Cardiology

Artificial intelligence has revolutionized various fields of medicine, including cardiology. With the advancements in technology, the use of AI in cardiology has become more sophisticated and its applications have widened. AI has the potential to improve the diagnosis, treatment management, and risk prediction of cardiac diseases, as well as the analysis of medical images such as echocardiograms or cardiac MRI scans.

Machine learning (ML) algorithms can be applied to clinical data, such as electrocardiograms (ECGs), echocardiograms, and medical imaging, to predict outcomes, stratify risk, and diagnose cardiovascular diseases. ML can also be used to identify patterns in data that may be invisible to the human eye, such as subtle changes in the heart’s electrical activity, and to develop personalized treatment plans.

Deep learning (DL) is a subset of ML that has revolutionized the field of medical-imaging analysis. DL models can analyze large amounts of cardiovascular images, such as computed tomography (CT) scans and magnetic resonance imaging (MRI) scans, and can detect abnormalities with high accuracy. DL can also be used to create 3D reconstructions of the heart from multiple 2D images, enabling detailed analysis of the heart’s structure and function.

Natural language processing technologies can be used to extract data from clinical documents such as electronic health records (EHRs) and physician notes, enabling the creation of large-scale clinical databases. NLP can also be used to develop algorithms that can identify and track specific cardiovascular risk factors, such as smoking, hypertension, and diabetes.

Computer-aided diagnosis (CAD) systems can be used to analyze medical images and provide diagnostic suggestions to clinicians. For example, CAD can be used to detect coronary artery disease by analyzing images of the coronary arteries and identifying areas of stenosis. CAD can also be used to analyze ECGs and identify abnormalities, such as arrhythmia.

Machine learning (ML) algorithms used in cardiology include the following:

1. The logistic regression algorithm is a type of regression analysis that is used for predicting the probability of a binary outcome. Logistic regression can be used in cardiology to predict the probability of a patient developing a cardiovascular disease based on various risk factors such as age, gender, and blood pressure.

Logistic regression models can be used to predict the likelihood of various cardiovascular events, including myocardial infarction (heart attack), stroke, and heart failure. Logistic regression models have been shown to be useful in identifying high-risk patients who may benefit from targeted interventions such as lifestyle modifications or medical therapy.

2. The decision trees algorithm is a machine learning method used for classification and regression analysis. In cardiology, decision trees can be used to create a model that can classify patients based on various cardiovascular risk factors, such as smoking status, blood pressure, and cholesterol levels.

Decision trees can be used to create decision support systems for clinicians to help them make more informed decisions about patient care. Decision trees can be used to identify patients who may be at high risk of developing cardiovascular disease and to develop personalized treatment plans for individual patients.

3. The random forest algorithm is an ensemble learning method that combines multiple decision trees to create a more accurate prediction model. In cardiology, random forests can be used to classify patients based on multiple decision trees, each tree using different combinations of risk factors.

Random forests can be used to create prediction models that are more accurate than individual decision trees. Random forests can identify the most important risk factors for cardiovascular disease and can help clinicians develop more effective treatment plans.

4. Support vector machines (SVMs)algorithms are supervised learning algorithms that are used for classification and regression analysis. In cardiology, SVMs can be used to identify patients at risk of developing cardiovascular disease by analyzing data such as age, blood pressure, and cholesterol levels.

SVM models can be used to identify patients at high risk of developing cardiovascular disease and to develop personalized treatment plans for these patients. SVM models can help clinicians make better-informed decisions about patient care and can improve patient outcomes.

Deep learning (DL) algorithms used in cardiology include the following:

1. Convolutional neural networks (CNNs) are a type of deep learning algorithm that is commonly used for image classification and recognition tasks. In cardiology, CNNs can analyze medical images such as CT scans, MRI scans, and echocardiograms, and identify various cardiovascular abnormalities such as heart disease and arrhythmia.

CNNs can be used to identify subtle changes in cardiac images that may indicate the presence of cardiovascular disease. CNNs can be used to develop more accurate and reliable diagnostic tools for clinicians, which can improve patient outcomes.

2. Recurrent neural networks (RNNs) are deep learning algorithms that are used for analyzing sequential data, such as time series. In cardiology, RNNs can analyze electrocardiogram (ECG) data and detect patterns and abnormalities that may indicate cardiovascular disease.

RNNs can be used to identify subtle changes in ECG data that may indicate the presence of cardiovascular disease. RNNs can be used to develop more accurate and reliable diagnostic tools for clinicians, which can improve patient outcomes.

Natural Language Processing (NLP) algorithms used in cardiology include the following:

1. Named entity recognition (NER) algorithms are NLP algorithms that are used to extract specific items such as diseases, symptoms, and medications from clinical documents such as electronic health records (EHRs). In cardiology, NER can be used to extract relevant information from patient records and help clinicians make better-informed decisions about patient care.

NER can be used to extract data from clinical documents such as EHRs and medical reports, which can be used to identify patterns and trends in patient data. NER can help clinicians identify patients who may be at high risk of developing cardiovascular disease and develop personalized treatment plans for these patients.

2. Sentiment analysis algorithms are NLP algorithms that are used to analyze text data and identify the sentiments or emotions conveyed by the text. In cardiology, sentiment analysis can be used to analyze patient feedback and reviews of cardiac treatments and procedures.

Sentiment analysis can be used to identify areas of patient care that may need improvement and to develop more patient-centered approaches to cardiac care. Sentiment analysis can help clinicians understand the patient experience and improve patient outcomes.

Data mining techniques used in cardiology include the following:

1. Association rule mining is a data mining technique that is used to identify patterns and relationships among different variables in a dataset. In cardiology, association rule mining can be used to identify risk factors and their relationships to various cardiovascular diseases.

Association rule mining can be used to identify complex patterns and relationships among different risk factors that may be missed by traditional statistical analysis. Association rule mining can help clinicians develop more effective risk prediction models and treatment plans for patients.

2. Clustering analysis is a data mining technique that is used to group similar data points together based on their characteristics. In cardiology, clustering analysis can be used to identify groups of patients with similar cardiovascular risk profiles.

Clustering analysis can be used to identify patient subgroups who may benefit from targeted interventions such as lifestyle modifications or medical therapy. Clustering analysis can help clinicians develop personalized treatment plans for individual patients and improve patient outcomes.

Computer-aided diagnosis (CAD) algorithms used in cardiology include the following:

1. Image segmentation is a type of algorithm used to separate an image into its component parts. In cardiology, image segmentation can be used to identify abnormalities in medical images, such as images of the heart and surrounding tissue.
2. Feature extraction is a type of algorithm used to identify specific features within an image. In cardiology, feature extraction can be used to identify specific features within medical images, such as the narrowing of coronary arteries.
3. Pattern recognition is a type of algorithm used to identify patterns in medical images such as the presence of plaques or blockages in arteries. In cardiology, pattern recognition can be used to identify patients who may be at risk of developing cardiovascular disease.

Examples of the usage of different AI technologies are briefly described in next section of this work.

### 5.2. Examples of Deployed Technologies

#### 5.2.1. Supervised Learning

Supervised learning had been found to be useful for many applications in cardiovascular medicine [26,34,35,36]. Moghaddasi et al. [37] created an SVM-based model for the detection of the severity of mitral regurgitation by video analysis of 2D echocardiography with 99.38% sensitivity, 99.63% specificity, and 99.45% accuracy. Attia et al. [38] used paired 12-lead electrocardiogram and echocardiogram data from 44,959 patients to train a convolutional neural network for the identification of patients with ventricular dysfunction. They tested a created network on an independent dataset of 52,870 patients that yielded results of 86.3% sensitivity, 85.7% specificity, and 85.7% accuracy. Porumb et al. [39] proposed an innovative approach to predict the occurrence of hypoglycemia based on electrocardiography. They trained two deep learning models—a convolutional neural network (CNN) and a convolutional neural network combined with a recurrent neural network (CNN + RNN)—to show that a hypoglycemic event can be automatically detected using electrocardiography. The received results presented as follows: CNN model—81.7% sensitivity, 87.5% specificity, and 82.4% accuracy; CNN + RNN model—84.7% sensitivity, 84.5% specificity, and 85.7% accuracy. Echocardiography images were used for developing automatic measurements of left ventricular strain. Salte et al. created a pipeline of an artificial neural network that was able to estimate motion as an alternative to traditional speckle-tracking-based measures of strain, successfully classifying cardiac views and performing the timing of cardiac events [40]. Another application of CNN was proposed by Kusunose et al. [41], who trained a model for the detection of regional wall motion abnormalities in echocardiography view, which yielded an AUC similar to that of cardiologists (0.99 vs. 0.98). 

Artificial neural networks have been successfully implemented for electrocardiogram interpretation. Their ability to find life-threatening arrythmias provides for many applications of artificial neural networks [42]. Galloway et al. [43] reported the possibility of enabling the usage of a trained CNN model to screen hyperkalemia in patients with renal disease from electrocardiograms, achieving an AUC above 0.85. Decision trees were found to be helpful in discriminating between patients with pulmonary vein drivers and those with extra pulmonary vein drivers of atrial fibrillation based on electrocardiogram, to aid in the identification of patients with high acute success rates due to pulmonary vein isolation [44]. A comparison approach of AI application for the diagnosis of acute coronary syndrome was undertaken by Berikal et al. [45]. The parallel training of four different algorithms yielded an advantage of SVM over ANN, NB, and logistic regression, with respective accuracies of 99.13%, 90.10%, 88.75%, and 91.26%. A decision trees algorithm variation—LogitBoost—was used by Motwani et al. [46] for the prediction of mortality in patients with coronary artery disease. The proposed model outperformed the standard Framingham risk score (AUC 0.79 vs. AUC 0.61). Machine learning algorithms were sown to be better than conventional statistical models used in everyday clinical practice for the discrimination of readmission and mortality of heart-failure patients [47]. In addition, as Kakadiaris et al. [15] reported, an SVM-based trained model outperformed an American College of Cardiology/American Heart Association (ACC/AHA) risk calculator by overlooking fewer cardiovascular disease (CVD) events and recommending fewer drug therapies. ANN algorithms enable the identification of high-risk patients after a myocardial infarction. This allows for the preparation of personalized therapy [48]. A gradient boosting model was applied by Kogan et al. [49] for the classification of patients with pulmonary hypertension using EHR. AI can be used for identifying predictors of acute coronary syndrome events or for risk stratification and the diagnosis of pulmonary arterial hypertension [13,16,50,51,52,53,54].

#### 5.2.2. Unsupervised Learning

Unsupervised learning was mainly used for clustering and grouping and as a preprocessing method for dimensional reduction. Karwath et al. [55] proposed the implementation of hierarchical clustering for distinguishing prognostic responses for β-blocker therapy in patients with heart failure and reduced the left ventricular ejection fraction. Cikes et al. [56] suggested that unsupervised learning algorithms can be used to combine standard clinical parameters and echocardiographic images to achieve an interpretable measure for clinicians’ classification of a phenotypical heterogeneous heart failure cohort and to identify patients who are more likely to respond to treatment. Unsupervised ML is still underrated in comparison to supervised ML, but it has been found to be an application in the analysis of EHR or genetic data for the automatization of data extraction [57,58]. An unsupervised algorithm called topological data analysis, used on combined EHR and genetic data, allowed Li et al. to reveal the presence of three separate subtypes of diabetes type 2 [59].

#### 5.2.3. Reinforcement Learning

The reinforcement learning state-action-reward-state-action (SARSA) algorithm performed a selection of dofetilide dose adjustments based on a negative reward for unsuccessful initiation [60]. Ghesu et al. [61] presented a novel method for real-time anatomical landmarks detection that was high in performance and robustness. Unfortunately, there are still only a few applications of reinforcement learning in cardiology. A possible niche for RL is the personalization of therapy for certain patient characteristics, because of the algorithm’s inherent decision-making construction.

#### 5.2.4. Natural Language Processing

NLP is a pre-processing method that makes native text understandable for machines. There are many nonmedicinal applications of natural language processing e.g., chatboxes and search engines. There is hope for more studies using NLP in the future, due to previously demonstrated improvement in algorithm performance with unstructured data [62,63,64,65,66,67,68]. Afzal et al. [69] recommended NLP as a tool for the rapid and efficient testing of peripheral arterial diseases based on clinical narrative notes. The NLP model outperformed billing code algorithms, yielding 91.2% sensitivity, 92.5% specificity, and 91.8% accuracy. Another example of improvement achieved by deploying NLP was reported by Ashburner et al. [70], whose study demonstrated that merging clinical and demographic features with incorporating narrative data from the EHR can greatly improve the efficiency of a model. Deploying NLP to studies may reduce misclassifications cause of the extraction of additional information available only in EHR.

Table 3 summarizes most of mentioned works and shows the main used algorithm. According to research by Jiang et al., two of the most-often-used AI algorithms for images, genetic data, and electrophysiology are SVMs and ANNs [71]. The wide application of SVMs in cardiovascular studies is dictated by their quite simple explainability of results and their ease of implementation. On the other hand, ANNs are usually very complex and they make it difficult to explain how a result was achieved; nevertheless, they have prominent computation power to handle image or electrophysiological data.

## 6. Discussion

Artificial intelligence is around us in every aspect of our lives. Smartwatches are no longer extraordinary and in addition to their many other features they can monitor health. Wearable technology can not only check how many calories have been burned or one’s actual heartbeat rate, they are also capable of detecting atrial fibrillation by applying an AI algorithm to differentiate the heart’s rhythm and provide recommendations to patients [72,73,74,75,76].

There is an urgent need to create official international requirements—legal, ethical, and methodical—for applying AI models to medicine [77,78,79]. Due to the rapid increase in the number of scientific articles about AI, some guidelines have already appeared, but many important questions remain unsettled [80,81,82]. Today, with growing computing power of super computers, we are able to analyze and process terabytes of data. The outcomes of such analyses should be used by physicians who can judge and reassess acquired information and, then, make decisions based on their knowledge, their experience, and, finally, AI suggestions. Machine learning might be able to overcome some of the limitations that occur in still-used statistical-based models. The challenges that must be overcome for the widespread use of AI in medicine can be divided into two groups: algorithm/model-related challenges and data-related challenges. The most important algorithm-related problems are standardization, reproducibility, explainability of predicted results, and legal and ethical responsibility for the outcome consequences for patients. Wrong assumptions and incorrect model-type selection may lead to faulty results and could be fatal for patients. The WHO has established ethical guidance for the implementation of artificial intelligence in healthcare [83].

The main challenges related to data involve the method of acquisition and safety. A highlighted case involving the transfer of patient-identifiable EHR without permission, from the Royal Free London NHS Foundation Trust to Google Deep Mind for algorithm development, has presented a major concern for public trust [84]. European regulations on data protection and privacy are well established after the publication of the General Data Protection Regulation by the European Union in 2016 [85]. 

Another tremendous challenge is related to data quality. The expression “garbage in, garbage out” precisely describes how poorly gathered and selected data can lead to erroneous assumptions and unsuccessful model predictions. It is crucial to develop international EHR registries that will not be outdated, biased, or discriminatory. A good EHR database should characterizes information with robustness, transparency, trust, and verifiability [86]. A solid and frequently checked security system must also be in place before full AI implementation [36]. The safety issue is not only related to data storing, but also to hacking resistance. The hackability of implanted medical devices is a life-threatening problem because of possible fatal consequences for patients. Recent research revealed the vulnerability of AI healthcare systems, including FDA warnings about the vulnerability of Medtronic insulin pumps to cyberattack [87,88,89].

**Figure 3:**
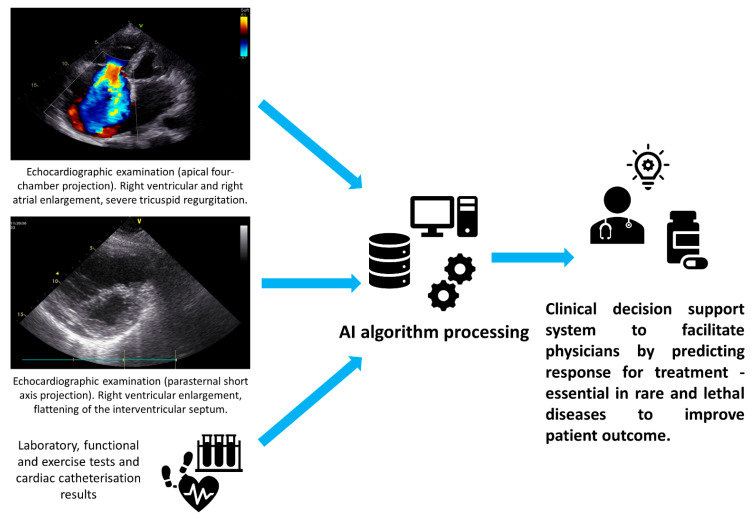
Future of clinical decision support systems.

The development of novel technologies is not only needed to benefit patients by improving diagnostic accuracy and providing personalized therapy that is focused on extending the quantity and quality of life. According to a Stanford University medicine report, there is an urgent need to help physicians with their work because of a high percentage of burnout, resulting in serious medical errors, inefficient patient care, and higher costs of care [90]. One of the reasons behind clinicians’ burnout is the vast amount of their work that is focused on preparing EHR—the time spent in preparing EHR is more than double the amount of time spent in front of patients. The loss of productivity was estimated to range from USD 90 billion to USD 140 billion [91]. Facilitating a clinical-decision process for physicians is essential, especially in the treatment of rare and lethal diseases. A great example is the future utilization of artificial intelligence to predict a pharmacotherapy response for pulmonary hypertension patients. See Figure 3. Such an approach could possibly improve the length and quality of life via personalized treatment. Utilizing a combination of AI and wearable technology to predict and manage cardiovascular therapy has the potential to usher in an era of precision medicine [92]. Undoubtedly, emerging machine learning approaches such as “Federated Learning”, which uses decentralized data and allows models to be trained on vastly distributed datasets based on patients across different clinics, will enable the training of highly accurate models for diagnosing cardiovascular diseases, while ensuring patient data privacy. However, this approach requires considerable further development [93].

## 7. Conclusions

Making use of artificial intelligence is crucial for future technological improvements. Medicine is facing increasingly new challenges and a global crisis, in the face of which machine learning has become a perfect complement to traditional forms of care and assistance. AI has many advantages that could be useful and helpful for physicians and patients. It also has some disadvantages and pitfalls, which everyone using AI-based technology should be aware of [94]. It is clear that artificial intelligence has the power to revolutionize the field of cardiology, offering new and innovative ways to diagnose, predict, and treat cardiovascular diseases. By leveraging AI technologies, we can improve the accuracy and efficiency of diagnosis, develop personalized treatment plans, and, ultimately, improve patient outcomes. While there are undoubtedly challenges, such as patient privacy, bias, and discrimination, that need to be addressed to ensure the responsible and ethical use of AI in cardiology, these concerns can be mitigated through the establishment of clear ethical guidelines and standards. By harnessing the power of AI to analyze complex data sets and identify patterns and trends, we have the potential to achieve more precise and effective cardiovascular care. Ultimately, the use of AI in cardiology offers a unique opportunity to transform the way we approach patient care and promote equitable healthcare outcomes, paving the way for a future of more personalized and effective cardiovascular medicine. In the words of Stephen Hawking, “Our future is a race between the growing power of technology and the wisdom with which we use it.”

## Figures and Tables

**Table 1:** Description of performance metrics for classification.

Performance Metric	Description
False positive (FP)	Object incorrectly classified as positive
False negative (FN)	Object incorrectly classified as negative
True positive (TP)	Object correctly classified as positive
True negative (TN)	Object correctly classified as negative
Precision (PPV)	The fraction of TP among all positive classified
Sensitivity (TPR)/recall	The fraction of TP that were correctly classified
accuracy	The fraction of TP and TN that were correctly classified
F1 score	The harmonic mean of precision and recall
Specificity (TNR)	The fraction of TN that were correctly classified
Receiver operating characteristic curve	The curve between recall (Y-axis) and =false positive Rate = 1-specificity (X-axis)
Area under the curve ROC	Evaluates the overall quality of the model
Precision recall curve	The curve between precision (Y-axis) and recall (X-axis)
Precision recall AUC (PR-AUC)	Alternative for AUC-ROC based on the PR curve

**Table 2:** Description of performance metrics for regression.

Performance Metric	Description
Mean absolute error (MAE)	The mean of the absolute difference between the actual and predicted values in a dataset
Mean squared error (MSE)	The mean squared error between the predicted and actual values in a dataset
Root mean squared error (RMSE)	The square root of MSE
Coefficient of determination R^2^	The proportion of variance explained by the model
Mean absolute percentage error (MAPE)	The mean of the absolute percentage errors of prediction

**Table 3:** Summarized studies and used algorithms.

Author	Study	Algorithm	Performance (Accuracy/AUROC/Precision)
Kogan et al. [49]	A machine learning approach to identifying patients with pulmonary hypertension using real-world electronic health records	XGBoost	0.92 AUROC
Mohammad et al. [48]	Development and validation of an artificial neural network algorithm to predict mortality and admission to hospital for heart failure after myocardial infarction: a nationwide population-based study	ANN	0.85–0.78 AUROC
Moghaddasi et al. [37]	Automatic assessment of mitral regurgitation severity based on extensive textural features on 2D echocardiography videos	SVM	99.45%
Attia et al. [38]	Screening for cardiac contractile dysfunction using an artificial intelligence-enabled electrocardiogram	CNN	85.70%
Porumb et al. [39]	Precision medicine and artificial intelligence: a pilot study on deep learning for hypoglycemic events detection based on ECG	CNN, RNN, Grad-CAM	82.40–85.70%
Salte et al. [40]	Artificial intelligence for automatic measurement of left ventricular strain in echocardiography	ANN	97–98%
Kusunose et al. [41]	A deep learning approach for assessment of regional wall motion abnormality from echocardiographic images	CNN	0.99–0.97 AUROC
Berikol et al. [45]	Diagnosis of acute coronary syndrome with a support vector machine	SVM, ANN, NB, Logistic Regression	90.10–99.13%
Motwani et al. [46]	Machine learning for prediction of all-cause mortality in patients with suspected coronary artery disease: a 5-year multicentre prospective registry analysis	DT—LogitBoost	0.79 AUROC
Kakadiaris et al. [15]	Machine learning outperforms ACC/AHA CVD risk calculator in MESA	SVM	0.92 AUROC
Kanwar et al. [50]	Risk stratification in pulmonary arterial hypertension using Bayesian analysis	Bayesian Network	0.80 AUROC
Galloway et al. [43]	Development and validation of a deep-learning model to screen for hyperkalemia from the electrocardiogram	CNN	0.85–0.88 AUROC
Luongo et al. [44]	Machine learning enables noninvasive prediction of atrial fibrillation driver location and acute pulmonary vein ablation success using the 12-lead ECG	DT	78.26%
Karwath et al. [55]	Redefining β-blocker response in heart failure patients with sinus rhythm and atrial fibrillation: a machine learning cluster analysis	Hierarchical clustering, Variational autoencoders (VAEs), K-means	N/A
Cikes et al. [56]	Machine learning-based phenogrouping in heart failure to identify responders to cardiac resynchronization therapy	K-means, Multiple Kernel Learning	N/A
Li et al. [59]	Identification of type 2 diabetes subgroups through topological analysis of patient similarity	Topological data analysis	N/A
Ghesu et al. [61]	Multi-scale deep reinforcement learning for real-time 3D-landmark detection in CT scans	Deep reinforcement learning	Accuracy improved by 20–30%
Levyid et al. [60]	Applications of machine learning in decision analysis for dose management for dofetilide	PCA, K-means, reinforcement learning—SARSA	86–93%
Garvin et al. [62]	Automating quality measures for heart failure using natural language processing: a descriptive study in the department of veterans affairs	NLP	Precision 98.7%
Shah et al. [63]	Impact of different electronic cohort definitions to identify patients with atrial fibrillation from the electronic medical record	NLP	0.89 AUROC
Kaspar et al. [64]	Underestimated prevalence of heart failure in hospital inpatients: a comparison of ICD codes and discharge letter information	NLP	Precision 96%
Patel et al. [65]	Development and validation of a heart failure with preserved ejection fraction cohort using electronic medical records	NLP	Precision 96%
Mahajan et al. [66]	Combining structured and unstructured data for predicting risk of readmission for heart failure patients	NLP	0.65 AUROC
Galper et al. [67]	Comparison of adverse event and device problem rates for transcatheter aortic valve replacement and mitraclip procedures as reported by the transcatheter valve therapy registry and the Food and Drug Administration postmarket surveillance data	NLP	N/A
Afzal et al. [69]	Mining peripheral arterial disease cases from narrative clinical notes using natural language processing	NLP	91.8%
Ashburner et al. [70]	Natural language processing to improve prediction of incident atrial fibrillation using electronic health records	NLP	N/A

## Data Availability

Not applicable.

## Abbreviations

AI	Artificial intelligence
ANN	Artificial neural network
AUC	Area under the curve
CNN	Convolutional neural network
DT	Decision tree
EHR	Electronic health records
FDA	Food and Drug Administration
GBT	Gradient-boosted tree
GradCAM	Gradient-weighted class activation mapping
HCP	Healthcare professionals
IDC	International Data Corporation
KNN	K-Nearest neighbor
ML	Machine learning
MLPDSC	Machine Learning for Pharmaceutical Discovery and Synthesis Consortium
NB	Naïve Bayes
NER	Unit name recognition
NLP	Natural language processing
PCA	Principal component analysis
RF	Random Forest
RL	Reinforcement learning
RNN	Recurrent neural network
SARSA	State-action-reward-state-action
SHAP	SHapley Additive exPlanations
SVM	Support vector machines
VAEs	Variational autoencoders
WHO	World Health Organization
XAI	Explainable artificial intelligence
